# The Side Effects of COVID-19 Vaccines and Its Association With ABO Blood Type Among the General Surgeons in Saudi Arabia

**DOI:** 10.7759/cureus.23628

**Published:** 2022-03-29

**Authors:** Mohammed Y Alessa, Fatimah J Aledili, Ahmad A Alnasser, Sarah S Aldharman, Abdulaziz M Al Dehailan, Hanan O Abuseer, Ali A Almohammed saleh, Hawra A Alsalem, Hassan M Alsadiq, Amal S Alsultan

**Affiliations:** 1 General Surgery, King Faisal University, Al Ahsa, SAU; 2 Medicine, King Faisal University, Al Ahsa, SAU; 3 Medicine, King Saud Bin Abdulaziz University for Health Sciences, Riyadh, SAU; 4 College of Medicine, King Faisal University, Al Ahsa, SAU; 5 Medicine, Taibah University, Medina, SAU; 6 Medicine, Imam Abdulrahman Bin Faisal University, Damam, SAU

**Keywords:** end, abo group, conception, conspiracy theory, side effect

## Abstract

Introduction

The benefits of vaccination outweigh its risks as it protects approximately two to three million individuals from infectious diseases annually. With the emergence of the coronavirus disease 2019 (COVID-19) pandemic, new vaccines have been developed. However, it is crucial to follow and recognize the side effects of COVID-19 vaccines. Previous studies have shown a relationship between ABO blood groups and coronavirus. Some vaccination side effects, such as muscle pain at the injection site and fatigue, may impair an individual's ability to perform tasks that require fine motor skills, such as those performed by a general surgeon. Therefore, this study aimed to identify the association between ABO blood groups and the side effects of COVID-19 vaccines among general surgeons in Saudi Arabia.

Method

A cross-sectional online survey-based study regarding the side effects following COVID-19 vaccination was conducted among Saudi and non-Saudi general surgeons working in public and private hospitals in Saudi Arabia who had received one or two doses of mRNA-based COVID-19 vaccines.

Results

A total of 612 surgeons responded. Approximately, 74.7% of the respondents reported side effects after receiving vaccines. Tiredness was the most commonly reported side effect of the vaccine, followed by severe local pain at the site of injection. Approximately, 16.2% of the participants started showing side effects 12 hours after receiving the vaccine. There was a significant relationship between the type of vaccine administered and the appearance of side effects (p = 0.004). The rate of appearance of side effects was higher in participants who received the Pfizer vaccine. However, there was no significant relationship between the appearance of side effects and age, gender, blood group, number of doses, and past history of COVID-19 infection (p > 0.05). Of the total participants, 256 (41.8%) stated that the side effects of the vaccine affected their work performance. Moreover, there was no significant difference in side effects, symptoms appearing after vaccination, the onset of symptoms, and duration of symptoms between the participants who received one dose and those who received two doses of the vaccine. In addition, there was no significant relationship between the severity of side effects and age, past history of COVID-19 infection, number of doses, and blood type (p > 0.05). However, there was a significant relationship between the severity of side effects and gender and type of vaccine (p = 0.000 and 0.004, respectively). A high percentage of females and those who received the AstraZeneca vaccine stated that their side effects affected their work performance.

Conclusion

Three-quarters of the participants reported side effects after the COVID-19 vaccination, which affected the work performance of 41% of participating general surgeons. There was no significant relationship between the appearance of symptoms and age, gender, blood group, number of doses, and past history of COVID-19 infection. However, there was a significant relationship between the severity of side effects and gender and type of vaccination. Future large-scale studies are recommended to further evaluate the implication of ABO blood type on COVID-19.

## Introduction

Following the World Health Organization's (WHO's) declaration of coronavirus disease 2019 (COVID-19) as a pandemic in March 2020, the Food and Drug Administration (FDA) allowed the emergency use of COVID-19 vaccination for individuals who are 16 years of age and older [1,2]. Since infections among healthcare workers (HCWs) have a serious outcome on patients, healthcare systems, and the general population, governments around the world develop vaccination strategies to prioritize their healthcare workers in getting vaccines to avoid the collapse of the healthcare system [2,3]. Almost every vaccination has side effects, but its benefits outweigh its risks. Vaccinations offset two to three million deaths from infectious diseases. However, adverse events must be identified and addressed promptly to minimize potential damage [4]. A cross-sectional study conducted in Poland found that the percentage of clinicians who were concerned about the long-term effects of COVID-19 vaccination was 32.14% [5]. Although the most often reported adverse effects are minor, such as pain at the injection site, tiredness, headache, and muscular discomfort, people still believe in the conspiracy theories and disinformation about COVID-19 vaccines [6,7]. A potential factor for the development of vaccine side effects is ABO blood groups. It has been found that blood group A was linked to a higher risk of COVID-19 infection, whereas blood group O was linked to a lower risk [8,9]. However, It is still not known whether the ABO blood group is related to the predilection of vaccine side effects or not. Therefore, this study aims to explore COVID-19 vaccine side effects and its association with the ABO blood group among general surgeons in Saudi Arabia. Also, we intend to identify the impact of these side effects on the performance of general surgeons.

Hypothesis

ABO blood groups can impact the side effects of general surgeons post-COVID-19 vaccination, and this affects the performance of general surgeons.

The rationale of the study

Researchers among healthcare workers in Saudi Arabia showed increasing numbers of individuals who suffered muscle pain at the site of injection, flu-like symptoms, fatigue, and other minor to moderate side effects after taking the COVID-19 vaccine [10]. Another study shows that there is an association between ABO blood groups and COVID-19 infection [9]. The surgeon's work necessitates manual dexterity and operative skills to perform an operation. COVID-19 vaccine side effects including extreme pain at the injection site, fatigue, and muscular discomfort may affect an individual's ability to perform tasks that require fine motor skills, such as those done by a general surgeon. This study addressed the following questions: Is there an association between COVID-19 vaccination side effects and ABO blood groups? Is the performance of the general surgeon affected by these side effects?

Objectives of the study

The primary objective of the study was to identify the side effects of the COVID-19 vaccine and its association with ABO blood groups among general surgeons in Saudi Arabia. Secondary objectives were to determine the effect of the vaccine's side effects on the surgeons’ work performance, compare the side effects and the number of doses received, and measure the association between demographics and these side effects.

## Materials and methods

A cross-sectional study was conducted in Saudi Arabia between July 2021 and May 2022. The target population was general surgeons (consultants, specialists, and residents) who worked in public or private hospitals in Saudi Arabia. Non-probability convenience sampling techniques have been used. A self-administered questionnaire was used to collect the data. An informed consent form was provided to all general surgeons who agreed to participate in the study. The questionnaire was provided electronically using Google Forms. The data were entered into Microsoft Excel (Microsoft Corporation, New Mexico, USA) and subsequently uploaded and analyzed using the Statistical Package for the Social Sciences (SPSS) software. Continuous variables such as age were reported as mean ± SD, whereas categorical variables such as gender were described using frequencies and percentages.

Inclusion criteria

Saudi and non-Saudi general surgeons of both genders (consultants, specialists, and residents) who worked in public or private hospitals in Saudi Arabia and received one or two doses of mRNA-based COVID-19 vaccine were included in this study.

Exclusion criteria

Those who did not receive the mRNA-based COVID-19 vaccine, other general population, and the participants who did not fill out the whole questionnaire were excluded from the study.

Study design and participants

This is a cross-sectional study, which is based on a survey questionnaire. We targeted Saudi and non-Saudi consultants, specialists, and residents in general surgery who work in public or private hospitals in different provinces in Saudi Arabia that have received one or two doses of the mRNA-based COVID-19 vaccine. The representative sample size required is 377, which was determined using the Richard Geiger equation with a margin error determined as 5%, confidence level determined as 95%, and the population determined as 20,000. The study duration is around 12 months.

Data collection instrument and procedures

The questionnaire from an online survey of symptoms following COVID-19 vaccination [3] in India, which consists of 15 questions that concentrate on the most common symptoms after receiving COVID-19 vaccines was used. Two optional questions were deleted, which were names and phone numbers. Four questions were modified - question number 1 in the first section and question numbers 1, 7, and 9 in the third section. Seven more questions were added to improve the study - question numbers 2 and 3 in the first section, question numbers 3-5 in the second section, and question numbers 8 and 11 in the third section to elicit more details from the participants.

The first page was designated for informed consent. An electronic google form survey was used and distributed on different social media platforms such as WhatsApp, Twitter, and Telegram. A modified English version of the referred questionnaire was used (Appendix 1). The questionnaire was validated by six experts: two experts from community medicine and public health, two physicians from internal medicine, and two immunity experts to ensure all items in the questionnaire were relevant for the study purpose. The developing questionnaire contained three sections that involved demographics, medical history, and vaccination side effects that might be encountered after each dose. Then, the experts were contacted via calls and emails and required to rate the relevance of each item in each section using a three-point Likert scale (1 = not relevant, 2 = somewhat relevant, 3 = relevant) and to suggest other items that might not have been considered. The final questionnaire was then piloted to ensure its coherence and wording.

Ethical consideration

All information was confidential and was only used for scientific research, and participation in this research was voluntary and optional with informed consent on the first page (Appendix 2). The data analysis and publication process did not require any identifiable personal data. The ethical approval of the study was obtained from the Ethical Committee of King Faisal University in Al Ahsa before initiating the study.

Statistical analysis

After extraction of the data, it was revised, coded, and fed to the statistical software SPSS version 26 (IBM Corp., Armonk, NY). All statistical analyses were done using two-tailed tests. Significance was adopted at p < 0.05 for the interpretation of the test results. The collected data of the participants were reported as the percentage of the categorical variables. The chi-square test was used to examine the association between categorical variables.

## Results

Demographic characteristics of the participants

The study included 650 participants of which four were excluded for not completing the questionnaire and 34 non-surgeons were excluded. The demographic characteristics of the participants are shown in Table 1.

**Table 1:** Demographic characteristics of the participants

Variables	N	%
Age group		
Below 50 years old	605	92.9%
Over 50 years old	46	7.1%
Sex		
Male	427	69.8%
Female	185	30.2%
Nationality		
Saudi	502	82.0%
Non-Saudi	110	18.0%
Level of training		
Resident	322	52.6%
Specialist	150	24.5%
Consultant	140	22.9%
Place of residency		
Eastern Province	156	25.5%
Makkah Province	108	17.6%
Riyadh Province	96	15.7%
Aseer Province	45	7.4%
Madinah Province	43	7.0%
Najran Province	27	4.4%
Qassim Province	26	4.2%
Tabuk Province	26	4.2%
Al-Baha Province	25	4.1%
Ha'il Province	22	3.6%
Northern Borders Province	20	3.3%
Al-Jawf Province	10	1.6%
Jazan Province	1	0.2%
Egypt	5	0.8%
Bahrain	1	0.2%
Spain	1	0.2%

History of COVID-19 infection and COVID-19 vaccine

Past History of COVID-19

Two hundred participants (32.7%) have a past history of COVID-19 infection, and 412 (67.3%) do not have a past history of COVID-19 infection. A total of 608 (99.3%) participants received the COVID-19 vaccine, while four (0.7%) did not receive the vaccine. Of those who received the vaccine, 103 (16.8%) received one dose, and 505 (82.5%) received two doses of the vaccine; 183 (30.1%) participants received AstraZeneca vaccine, 400 (65.8%) received Pfizer, and 25 (4.1%) received both.

Side Effects of COVID-19 Vaccine

**Figure 1:**
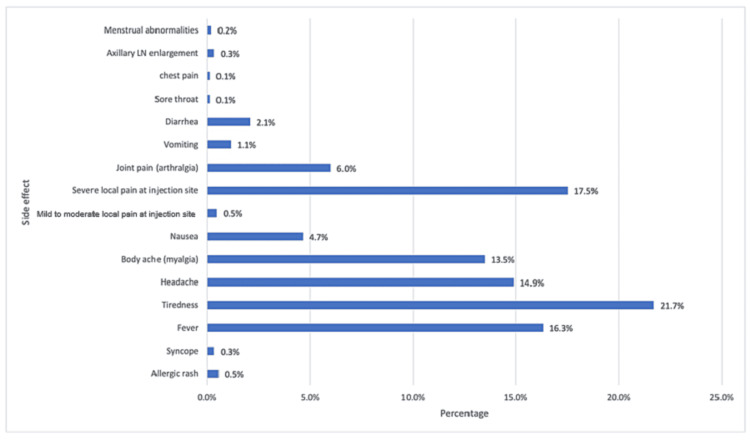
Side effects of the COVID-19 vaccine among the participants

A total of 454 participants (74.7%) had symptoms after receiving the vaccine, and 154 (25.3%) had no symptoms. Tiredness was the most common side effect of the vaccine, followed by severe local pain at the site of the injection. Figure 1 illustrates the side effects that the participants had after receiving the vaccine. In 99 participants (16.2%), the symptoms started 12 hours after receiving the vaccination; in 84 (13.7%), it started after six hours; in 82 (13.4%), it started after two hours; in 79 (12.9%), it started after four hours; in 76 (12.4%), it started after 24 hours, and in 47 (7.7%), the symptoms started eight hours after receiving the vaccine. Regarding the duration of symptoms, 136 participants (22.2%) had symptoms that last for 24 hours, 110 (18%) had symptoms that last for 48 hours, 69 (11.3%) had symptoms that last for 12 hours, 56 (9.2%) had symptoms that last for more than 48 hours, 53 (8.7%) had symptoms that last for six to eight hours, 24 (3.9%) had symptoms that last for four hours, and 18 (2.9%) had symptoms that last for two hours only.

Using the chi-square test, a significant relationship was found between the type of vaccine and the appearance of symptoms (p-value = 0.004), in which the percentage of participants with symptoms was higher in those who received the Pfizer vaccine. However, there was no significant relationship between the age range, gender, blood group, number of doses, and past history of COVID-19 infection (p-value > 0.05).

Effect of the vaccine on the work performance of surgeons

**Figure 2:**
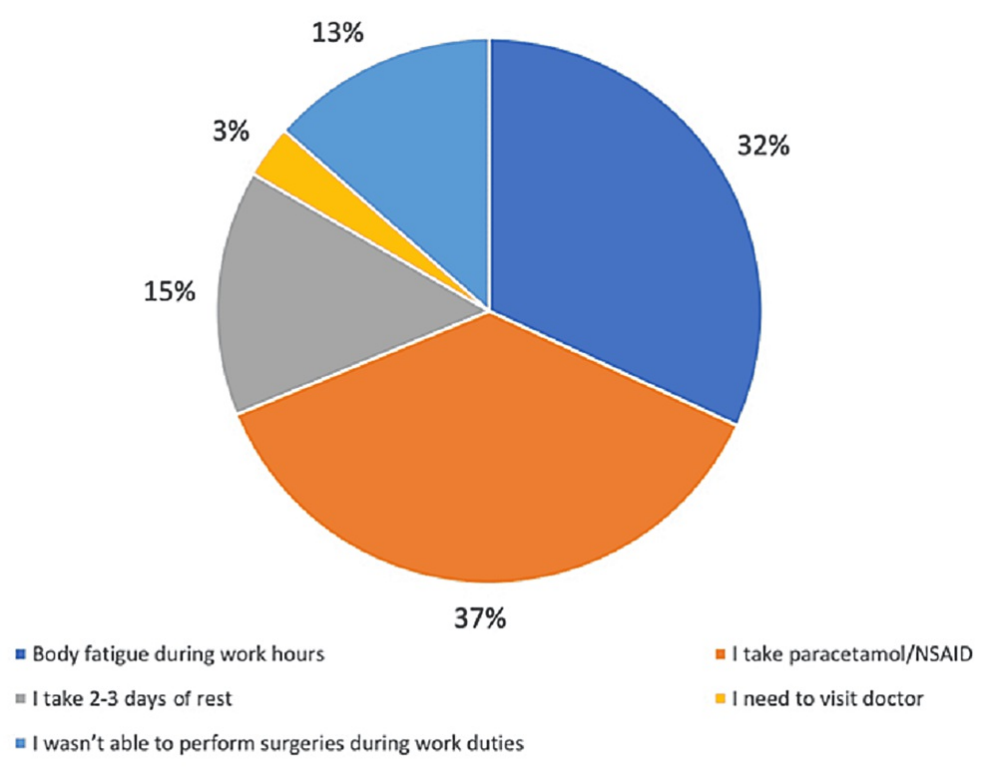
The effect of COVID-19 vaccine on the work performance of the surgeons

A total of 256 participants (41.8%) stated that the vaccine's side effects affected their work performance, and 356 (58.2%) stated that it did not affect their work performance. It was found that 200 participants (36.9%) needed to take paracetamol or nonsteroidal anti-inflammatory drugs (NSAIDs), 173 (31.9%) had body fatigue during work hours, 79 (14.6%) needed to take two to three days of rest, 73 (13.5%) were not able to perform surgeries during work hours, and 17 (3.1%) needed to visit a doctor. Figure 2 illustrates the effect of the COVID-19 vaccine on the surgeons’ work performance.

Taking paracetamol or NSAIDs was helpful for 406 participants (66.3%), while it was not helpful for 77 participants (12.6%). However, 129 participants (21.1%) did not take paracetamol or NSAIDs. Regarding the expectation of the surgeons on the immune system’s response to the vaccine, 402 (65.7%) stated that the response matches their expectations, while 142 (23.2%) stated that their response did not match their expectation in which they expected more symptoms, and 68 (11.1%) stated that the symptoms were worse than they thought.

Factors affecting the severity of the vaccine's side effects

Using the chi-square test, a significant statistical relationship was found between gender, type of vaccine, and severity of COVID-19 vaccine's side effects (p-values = 0.000 and 0.004), in which a high percentage of females and the participants who received the AstraZeneca vaccine stated that their symptoms affected their work performance. However, there was no significant relationship between age range, past history of COVID-19 infection, number of doses, and blood type with the severity of the vaccine's side effects (p-value > 0.05). Table 2 illustrates the relationship between the severity of side effects associated with COVID-19 vaccination and various factors.

**Table 2:** Factors affecting the severity of side effects associated with COVID-19 vaccination P: Pearson's X^2^ test. *p-value < 0.05 (significant).

Variables		Did the symptoms affect your work?				p-value
		No		Yes		
		No	%	No	%	
Age group	Less than 50 years old	353	58.4%	251	41.6%	0.438
	More than 50 years old	30	65.2%	16	34.8%	
Gender	Male	270	63.2%	157	36.8%	0.000*
	Female	86	46.5%	99	53.5%	
Past history of COVID-19 infection	No	245	59.5%	167	40.5%	0.352
	Yes	111	55.5%	89	44.5%	
Type of vaccine received	Not vaccinated	1	100.0%	0	0.0%	0.014*
	AstraZeneca	90	48.9%	94	51.1%	
	Pfizer	250	62.5%	150	37.5%	
	Both	15	55.6%	12	44.4%	
Number of doses	One dose	59	57.3%	44	42.7%	0.832
	Two doses	295	58.4%	210	41.6%	
Blood type	Type A	83	51.60%	78	48.40%	0.241
	Type B	77	58.80%	54	41.20%	
	Type O	157	61.60%	98	38.40%	
	Type AB	39	60.00%	26	40.00%	

Comparison between the side effects of the vaccine and the number of doses received

**Figure 3:**
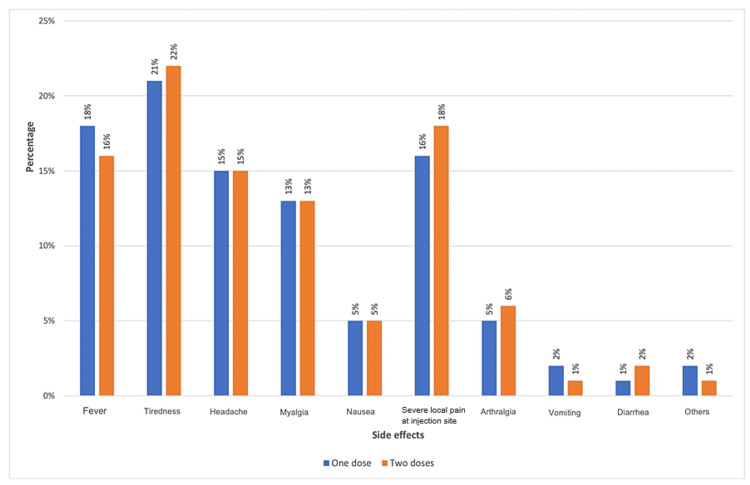
Comparison between the vaccine's side effects and the number of doses received

Using the chi-square test, no significant difference was found between the side effects, symptoms appearing after vaccination, onset of symptoms, and duration of the symptoms (p-value = 0.259, 0.189, 0.236, and 0.381, respectively) among the participants who received one or two doses. Figure 3 and Table 3 illustrate the difference in receiving different numbers of doses.

**Table 3:** The effect of the difference in the number of doses P: Pearson's X^2^ test. p-value < 0.05 (significant).

Variables		One dose		Two doses		p-value
Symptoms appearing after vaccination	No	32	20.40%	125	79.60%	0.189
	Yes	71	15.70%	380	84.30%	
Onset of symptoms	2 hours	9	11.10%	72	88.90%	0.236
	4 hours	8	10.30%	70	89.70%	
	6 hours	17	20.20%	67	79.80%	
	8 hours	7	14.90%	40	85.10%	
	12 hours	21	21.40%	77	78.60%	
	24 hours	12	15.80%	64	84.20%	
Duration of the symptoms	2 hours	4	23.50%	13	76.50%	0.381
	4 hours	3	12.50%	21	87.50%	
	6–8 hours	7	13.20%	46	86.80%	
	12 hours	8	11.80%	60	88.20%	
	24 hours	23	16.90%	113	83.10%	
	48 hours	14	12.70%	96	87.30%	
	More than 48 hours	14	25.50%	41	74.50%	

## Discussion

Vaccines provide a significant public health advantage. They may be the most practical method to put an end to social distancing in the event of COVID-19. Vaccines, like any medication, can cause side effects that may have a negative outcome on healthcare workers, especially general surgeons who spend more hours doing surgeries. To the best of our knowledge, this is a rare study that assesses the side effects of the COVID-19 vaccine among general surgeons in Saudi Arabia. In this study, the data collected revealed that most of the side effects appeared after the second dose (84.30%). This finding is in accordance with a study in the Czech Republic [6]. The current study showed that the most common side effect is tiredness (21.70%), followed by severe local pain at the injection site (17.60%), followed by fever (16.40%). This is in contrast with a recent Saudi study conducted on residents [10].

In our research, the main objective was to identify the side effects of the COVID-19 vaccine and its association with ABO blood groups, and their effect on general surgeons in Saudi Arabia. Based on the results, general surgeons who received the COVID-19 vaccine experienced the following symptoms: tiredness - being the most common - followed by pain at the injection site, fever, headache, and myalgia. This correlates with the cross-sectional survey-based research that was conducted in the Czech Republic to gather data on the side effects of the COVID-19 vaccine among healthcare workers [6]. The most often reported adverse effects in this study were injection site discomfort, tiredness, headache, a muscular discomfort [11]. Also, almost the same symptoms were found in a systematic review study that was conducted to consolidate the evidence on the safety data from the published COVID-19 vaccine trials done between December 2019 and 2020 [11]. The majority of the reactions recorded were mild to moderate in severity, with a few exceptions being severe. All reactions subsided after three to four days. Pain at the injection site, swelling, and redness were the most often reported local adverse effects [11]. Another study was conducted to identify the COVID-19 vaccine's adverse effects among the hospital staff at an Indonesian national referral hospital. A total of 840 responses were gathered in this study. The adverse effects included swelling, fever, exhaustion, diarrhea, dyspnea, anaphylactic response, and enlarged lymph nodes [12]. In conclusion, adverse effects such as tiredness, headache, pain at the site of injection, and fever were significant findings in all of these studies, so it can be expected in all the individuals who are receiving the vaccine.

Based on our results, there was also a significant relationship between gender and the severity of the COVID-19 vaccine's side effects, with a high proportion of females reporting that their symptoms interfered with their work performance. This was also the finding of a retrospective cross-sectional study conducted to track the vaccine's short-term adverse effects among Saudi residents [10]. The study found that the number of females who experienced vaccine side effects was significantly higher than the number of males [10].

On the other hand, our study revealed that the relationship between ABO blood groups and the COVID-19 vaccine's side effects is still insignificant. Research among a bigger sample size and a less specified group should be done to address this relationship. The idea for this question arose after reading research that looked at the relationship between the ABO and Rh blood types and infection, intubation, and mortality in 14,112 subjects who had been tested for severe acute respiratory syndrome coronavirus 2 (SARS-CoV-2) and had a known blood type at the New York-Presbyterian (NYP) Hospital system [13]. It was found that non-O types had a slightly higher infection prevalence. When compared with blood types, the risk of intubation was lower for type A and higher for types AB and B, whereas the risk of mortality was higher for type AB and lower for types A and B. They found that having Rh-negative blood had a protective role on all three outcomes [13]. This was supported by other studies such as a retrospective research conducted in China to investigate the relationship between the blood type distribution and SARS-CoV-2 infection, progression, and outcome in COVID-19 patients [14]. The blood type A population was shown to be more sensitive to SARS-CoV-2 in a study of 265 individuals from different medical sites and two established cohorts [14].

Limitations

Our study has limitations as this is a cross-sectional study among general surgeons in Saudi Arabia, which does not include all healthcare providers or the general population. However, it gives a preview of the side effects associated with COVID-19 vaccination in our target population. Also, other vaccines that are not mRNA-based COVID-19 vaccines were not included in this study.

Recommendations

Understanding the relationship between COVID-19 vaccine side effects and ABO blood types is important for determining the influence of these side effects on the performance of healthcare professionals. Further studies are recommended to confirm the relationship between the COVID-19 vaccine side effects and ABO blood groups taking into consideration all healthcare providers as well as the general population. We also recommend studying other COVID-19 vaccines and looking into more variables that may affect the severity of side effects associated with COVID-19 vaccination.

## Conclusions

Almost all participants received COVID-19 vaccines, and more than half of them received two doses of the vaccine. Three-quarters of participants reported side effects after the COVID-19 vaccination, which affected the work performance of 41% of participating general surgeons. Tiredness was the most common side effect of the vaccine, followed by severe local pain at the injection site. There was a significant relationship between the type of vaccine and the appearance of symptoms in which the percentage of participants who had symptoms was higher in those who received the Pfizer vaccine. There was a significant relationship between gender, type of vaccination, and severity of COVID-19 vaccine's side effects, with a high proportion of females and the participants who received AstraZeneca vaccine reporting that their symptoms interfered with their work performance. However, there was no significant relationship between the appearance of symptoms and age range, gender, blood group, number of doses, and past history of COVID-19 infection. Further studies on a larger scale are recommended to further evaluate the implication of ABO blood type on COVID-19 vaccination.

## Appendices

Appendix 1: English version of the COVID-19 vaccine's side effects and its association with ABO blood type among general surgeons questionnaire in Saudi Arabia

First Section

1. Are you a surgeon?

- Yes

- No

2. Did you take the COVID-19 vaccine?

- Yes

- No

3. If yes, how many doses?

- One dose

- Two doses

Second Section: Biographical Data

1. Age group? (in years)

- Less than 50 years 

- More than 50 years 

2. Sex?

- Male

- Female

3. Nationality?

- Saudi

- Non-Saudi

4. Where are you from?

- Riyadh Province

- Makkah Province

- Eastern Province

- Madinah Province

- Al Baha Province

- Al Jawf Province

- Northern Borders Province

- Qassim Province

- Ha'il Province

- Tabuk Province

- Aseer Province

- Jazan Province

- Najran Province

- None of the above … Please write it (.................)

5. What is your level of training?

- Consultants

- Specialists

- Residents

Third Section 

1. Which vaccine? 

- Pfizer

- AstraZeneca

- Others (please write)

2. Past history of COVID-19?

- Yes

- No

3. Any symptoms following the vaccine?

- Yes

- No

4. If yes, the onset of symptoms within?

- 2 hours 

- 4 hours

- 6 hours

- 8 hours

- 12 hours

- 24 hours

5. How long did the symptoms last?

- 2 hours

- 4 hours

- 6 hours

- 8 hours

- 12 hours

- 24 hours

- 48 hours

- More than 48 hours

6. What were the symptoms? (Multiple answers are allowed)

- Fever

- Tiredness

- Headache

- Severe local pain at the injection site 

- Body ache (myalgia)

- Joint pain (arthralgia)

- Nausea

- Vomiting

- Diarrhea

- Syncope

- Allergic rash

- Others

7. Did the symptoms affect your work?

- Yes

- No

8. If yes, how was that? (Multiple answers are allowed)

- Body fatigue during work hours

- I was not able to perform surgeries during work hours

- I take paracetamol/NSAID

- I take two to three days of rest 

- I need to visit a doctor 

9. Was taking paracetamol or NSAID helpful?

- Yes

- No

- I did not take paracetamol or NSAID

10. Did the symptoms match your expectation of the immune system’s response to the vaccine?

- Yes (I am happy that my body responded)

- No (I expected more symptoms)

- Worse than I thought

11. What is your ABO blood type?

- Type A

- Type B

- Type O

- Type AB

Appendix 2: Consent form

(Informed Consent)

We are a group of researchers from the Faculty of Medicine, we invite you to participate in a scientific research entitled "The Side Effects of COVID-19 Vaccines and Its Association With ABO Blood Type Among the General Surgeons In Saudi Arabia" to learn about ABO blood groups and their association with COVID-19 vaccine's side effects and how the side effects affected general surgeons' jobs.

♦ The questionnaire consists of the following sections:

First: Informed consent.

Two: Biographic data.

Third: Special questions for practicing physicians in the field of general surgery (Consultants, Specialists, and Residents) who work in public or private hospitals in Saudi Arabia that have received one or two doses of the mRNA-based COVID-19 vaccine.

Do you agree to participate in the research?

- Yes

- No

*Noting that all information will be confidential and will only be used for the purpose of scientific research and that participation in this research is voluntary and optional and will only require three to four minutes to fill out the questionnaire completely, with the right to withdraw at any time.

For comments, the principal investigator, Dr. Mohammed Alessa, can be contacted at the following e-mail: .
